# Tofacitinib for the Treatment of Severe Interstitial Lung Disease Related to Rheumatoid Arthritis

**DOI:** 10.1155/2021/6652845

**Published:** 2021-04-22

**Authors:** Caterina Vacchi, Andreina Manfredi, Giulia Cassone, Stefania Cerri, Giovanni Della Casa, Dario Andrisani, Carlo Salvarani, Marco Sebastiani

**Affiliations:** ^1^PhD Program in Clinical and Experimental Medicine, University of Modena and Reggio Emilia, Via Del Pozzo 71, Modena 41125, Italy; ^2^Rheumatology Unit, University of Modena and Reggio Emilia, Via Del Pozzo 71, Modena 41125, Italy; ^3^Respiratory Diseases Unit and Centre for Rare Lung Diseases, University of Modena Reggio Emilia, Via Del Pozzo 71, Modena 41125, Italy; ^4^Radiology Unit, University of Modena and Reggio Emilia, Via Del Pozzo 71, Modena 41125, Italy

## Abstract

Rheumatoid arthritis (RA) is a chronic systemic inflammatory disease characterized by chronic symmetrical erosive synovitis and extra-articular manifestations, including interstitial lung disease (ILD), whose treatment is nowadays challenging due to high infectious risk and possible pulmonary iatrogenic toxicity. Janus kinase inhibitors, namely, tofacitinib, baricitinib, and upadacitinib, are the latest drug class for the treatment of RA with a good safety profile. We present the case of a patient with RA-ILD successfully treated with tofacitinib. A 52-year-old man was referred to our multidisciplinary clinic for rheumatic and pulmonary diseases for an active erosive seropositive RA and progressive ILD. Previous treatments were GC, hydroxychloroquine, methotrexate, etanercept, withdrawn after ILD detection, and tocilizumab, discontinued due to relapsing infections. After our evaluation, we proposed rituximab in addition to low-dose GC and hydroxychloroquine, ineffective on joint involvement. Therefore, we proposed tofacitinib which allowed us to control joint involvement, stabilize ILD improving respiratory symptoms, and manage the frequent infectious episodes that occurred initially. The short half-life and rapid-acting of tofacitinib are two helpful characteristics regarding this aspect. Despite limited data from randomized trials and real-life, tofacitinib could represent a safe therapeutic option for RA-ILD patients. Longitudinal studies are required to confirm this encouraging report.

## 1. Introduction

Rheumatoid arthritis (RA) is a chronic systemic inflammatory disease affecting 0.5–1% of the population worldwide. It is characterized by chronic symmetrical erosive synovitis and sometimes by extra-articular manifestations [1], including interstitial lung disease (ILD), which represents the most common lung involvement [2]. ILD significantly affects therapeutic approach, quality of life, morbidity, and mortality of RA patients, with an estimated prevalence ranging from 4 to 30% [2, 3].

Among the main risk factors for the development of AR-ILD, we consider high titles of rheumatoid factor (RF) and anticitrullinated protein antibodies (ACPA), male sex, and cigarette smoking history [4]. Sometimes lung interstitial involvement is early and represents the only sign of disease. In these cases, a clinical and serological follow-up may lead to evidence of autoimmune disease later in time [5].

In AR patients with lung involvement, the radiological UIP pattern is not only the most frequent one (about 60–70%) but also the most associated with poor prognosis [1, 6].

The treatment of patients with RA and concomitant ILD is nowadays challenging and mainly based on glucocorticoids (GC) and immunosuppressants [7]. Therapeutic options in these patients are complicated by the possible pulmonary toxicity of many disease modifying antirheumatic drugs (DMARDs) and by their unclear efficacy on pulmonary involvement [7]. The scientific community is constantly looking for therapeutic alternatives to control RA activity avoiding lung toxicity and, possibly, influencing the natural history of RA-ILD. Furthermore, RA-ILD treatment should be safe and easy to manage in case of infectious adverse events, giving the higher infectious risk in ILD patients [8].

Small-molecule Janus kinase inhibitors (JAK-i), namely, tofacitinib, baricitinib, and upadacitinib, are the latest drug class of DMARDs commercialized for the treatment of RA with a good safety profile [9–13].

Here, we present the case of a patient affected with progressive RA-ILD successfully treated with tofacitinib.

## 2. Case Report

A 52-year-old man, former smoker (45 pack years), was referred to our multidisciplinary outpatient clinic for rheumatic and rare lung diseases for an active RA associated with progressive ILD. His past clinical history revealed systemic arterial hypertension, dyslipidaemia, nonalcoholic fatty liver disease, and class II obesity (BMI: 37).

Diagnosis of RA was performed in April 2012. RF and ACPA were positive (42 and 300 U/mL, respectively), while antinuclear antibodies (ANA), extractable nuclear antigens (ENA), and antineutrophil cytoplasmic antibodies (ANCA) were negative.

Arthritis was aggressive and rapidly erosive at hands and wrists. The patient had been initially treated with methotrexate (MTX) 15 mg weekly, effective but replaced with hydroxychloroquine (HCQ) 400 mg daily for the desire of paternity in December 2012. Since HCQ became ineffective, etanercept (ETA) 50 mg weekly was added in April 2014.

**Figure 1:**
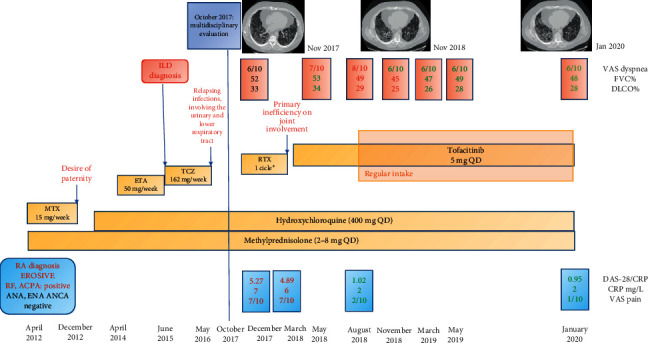
Clinical course and therapeutic strategy. ILD: interstitial lung disease; MTX: methotrexate; ETA: etanercept; TCZ: tocilizumab; RTX: rituximab; VAS: Visual Analogue Scale; FVC: forced vital capacity; DLCO: diffusing capacity of the lung for carbon monoxide; RA: rheumatoid arthritis; CRP: C-reactive protein; RF: rheumatoid factor; ACPA: anticitrullinated protein antibodies; ANA: antinuclear antibodies; ENA: extractable nuclear antigen; ANCA: antineutrophil cytoplasmic antibodies; DAS-28: disease activity score on 28 joints.

Fourteen months later, ILD was casually detected (Figure 1), and ETA was replaced with subcutaneous tocilizumab (TCZ) 162 mg weekly. This latter drug was stopped after few months for relapsing infections, mainly involving the urinary and lower respiratory tract (Figure 1).

In October 2017, due to a deterioration of respiratory symptoms, he was referred for the first time to our multidisciplinary outpatient clinic, which includes a rheumatologist and a pulmonologist. At that time, he presented severe restrictive ventilatory impairment, with a forced vital capacity (FVC) of 52% of the predicted value and a severe reduction in gas exchange with a diffusion capacity for carbon monoxide (DLCO) of 33% of the predicted value. Moreover, he desaturated at 6-minute walking test (nadir reached after two minutes with an oxygen saturation of 87%). As far as RA activity, he showed arthritis of wrists and II-III right metacarpophalangeal; C-reactive protein (CPR; 50 mg/L) and erythrocyte sedimentation rate (ESR; 50 mm/h) were both increased, with a 28-joint disease activity score (DAS-28 CRP) of 5.27.

**Figure 2:**
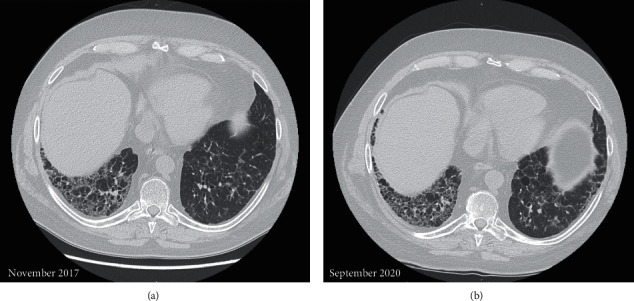
Chest high-resolution computed tomography: November 2017 compared to last follow-up, September 2020. Reticular abnormalities, honeycombing aspects, and traction bronchiectasis compatible with usual interstitial pneumonia. Despite an initial progression, interstitial lung disease showed a substantial stability at the last follow-up.

At chest high-resolution computed tomography (HRCT), a pattern of usual interstitial pneumonia was described, characterized by reticular abnormalities and honeycombing aspects, particularly at the right lower lobe (Figure 2).

At that time, he was taking HCQ and low-dose prednisone (5 mg daily).

According to the clinical picture, we prescribed oxygen supplementation and proposed treatment with rituximab (1000 mg every other week for 2 intravenous infusions), performed in December 2017, maintaining treatment with prednisone and HCQ. Two months later, arthritis was still active, involving bilateral MCF and proximal interphalangeal joints of hands, wrists, and ankles associated with ESR and CRP elevation (45 mm and 60 mg/L, respectively).

After multidisciplinary discussion with a pulmonologist, in March 2018, we prescribed tofacitinib 5 mg twice daily. Vaccination for varicella zoster virus (VZV) was not performed because of the immunosuppressive condition of the patient.

One month later, the patient presented an infection of the upper respiratory tract, successfully treated with antibiotic therapy; tofacitinib was discontinued for ten days. Despite other few respiratory and urinary infectious episodes, RA remission was obtained after 3 months. Disease flares appeared when tofacitinib was discontinued, but every time, a rapid arthritis control was obtained with the reintroduction of tofacitinib.

From August 2018 to March 2020, we observed a stable RA remission, and no other infections were reported by the patient.

Despite an initial radiological progression detected at HRCT in November 2018, respiratory symptoms improved and lung function (FVC and DLCO) remained stable over the time (Figure 1).

## 3. Discussion

ILD is a serious pulmonary complication of RA characterized by a significant impact on morbidity and mortality, representing a current therapeutic challenge because of the possible pulmonary toxicity of many traditional and biological DMARDs, their unclear efficacy on pulmonary disease, and the higher infectious risk in comparison to non-ILD RA patients [7, 8].

JAK-is have recently emerged in clinical practice for the treatment of RA, and they are recommended by the European League against Rheumatism (EULAR) in patients failing an initial treatment with MTX or other conventional DMARDs with poor prognostic factors [14].

Despite limited data, no relevant safety concerns in relation to both ILD onset and progression are emerged for tofacitinib [9, 11, 15, 16].

A post hoc analysis on 7061 RA patients receiving tofacitinib from clinical trials and long-term extension studies reported an incidence rate for ILD with both tofacitinib doses of 0.18 per 100 patient-years [17].

Few real-life data are up to now available. Among 15 RA-ILD patients treated with tofacitinib, none showed worsening of dyspnoea, while improvement was reported in some cases; moreover, respiratory function and DLCO remained stable in the majority of subjects evaluated, while 4 patients improved [18].

In another study, 3 RA-ILD patients were successfully treated with tofacitinib without pulmonary adverse events in an 8–12-month period [19].

About the possible effect on ILD, some case reports have recently been published in which tofacitinib appears to be effective on ILD related to antimelanoma differentiation-associated 5 gene antibody-positive dermatomyositis and antisynthetase syndrome [20–23].

Tofacitinib has been demonstrated to be able to reduce the progression of zymosan-induced ILD in SKG mice, a murine model of RA. Tofacitinib significantly suppressed the progression of ILD compared to control by expanding myeloid-derived suppressor cells and suppressing Th17 cells' proliferation and differentiation, suggesting a potential therapeutic effect of tofacitinib for RA-ILD [24].

However, other in vitro studies reported that the inhibition of JAK2, but not the selective JAK1/JAK3 pathway, significantly reduced IL-17A-induced fibrogenic response in fibroblast from IPF and RA-ILD patients [25].

Rituximab was proposed as a therapeutic option for RA-ILD, even if it has been associated with an increased risk of infections in patients with RA-ILD.

Unfortunately, in our patient, rituximab was ineffective on arthritis and ILD, and it could have contributed to the infectious adverse events showed by the patient in the next six months [26].

The short half-life and rapid-acting of tofacitinib are two characteristics that we have considered for the treatment of our patient. Moreover, we took advantage by an intermittent therapy when the infectious events appeared obtaining a rapid clearance of the drug. On the other side, the patient also showed a rapid flare of disease after tofacitinib discontinuation, requiring a temporary increase of prednisone dose.

## 4. Conclusion

Despite limited data from clinical trials and real-life, tofacitinib could represent a therapeutic option for RA-ILD patients, with a good safety profile. Longitudinal studies are required to confirm this encouraging report.
